# Retraction: mTOR up-regulation of SNRPA1 contributes to hepatocellular carcinoma development

**DOI:** 10.1042/BSR20193815_RET

**Published:** 2025-12-18

This article is being retracted from *Bioscience Reports* at the request of the Editor-in-Chief and the Editorial Board. This follows the receipt of a notification from a reader, alerting the Editorial Board to similarities that exist between an image in this article and those in two other articles.

Specifically, the Figure 2D SNRPA1 bands appear similar to the Figure 6C AKT bands from Gong et al. 2015 (doi: 10.18632/oncotarget.4624) and to the Figure 2D GOLIM4 bands from Bai et al. 2018 after a vertical flip (https://doi.org/10.1042/BSR20180454). The authors were contacted and provided repeated experimental data for the western blots. However, they were unable to provide original data.

The Editorial Board therefore has significant concerns surrounding the validity of the data and stands by the decision to retract the article. The authors did not respond when contacted regarding the retraction.

